# Editorial: The role of autophagy in cardiovascular disease

**DOI:** 10.3389/fcell.2025.1697218

**Published:** 2025-11-19

**Authors:** Qinchun Duan, Xuehong Xu, Odell D. Jones, Jianjie Ma, Joseph L. Bryant, MengMeng Xu

**Affiliations:** 1 Laboratory of Cell Biology, Genetics and Developmental Biology, College of Life Sciences and University Hospital, Shaanxi Normal University, Xi’an, China; 2 University Laboratory Animal Resources (ULAR), University of Pennsylvania School of Medicine, Philadelphia, PA, United States; 3 Division of Surgical Sciences, Department of Surgery, University of Virginia Medical School, Charlottesville, VA, United States; 4 Institute of Human Virology, University of Maryland School of Medicine, Baltimore, MD, United States; 5 Department of Pediatrics, Morgan Stanley Children’s Hospital, Columbia University, New York, NY, United States

**Keywords:** cardioprotective function of autophagy, endocardial and myocardial precursors, cellular conduction system of tunneling nanotube TNT, autophagy associated physiological homeostasis, selective and non-selective autophagy in heart

## Introduction

As autophagy a highly evolutionary conserved catabolic process, autophagy plays a critical role in maintaining cellular quality control across species, which also links cardiac development to cardiac pathogenesis, from *Drosophila* to humans. During cardiac organogenesis, autophagy is essential for preserving cardiac homeostasis by eliminating dysfunctional organelles, misfolded proteins, and damaged macromolecules. Genetic manipulation in *Drosophila* has provided a foundational understanding for the importance of autophagy in cardiac development. Studies in human aging and pathogenic process has also linked this vital cellular process to stress, aging, and metabolic challenges to offer a unifying molecular and cellular framework underlying cardiac pathogenesis.

## Insights from *Drosophila melanogaster*: the genetic foundation of cardiac autophagy

*Drosophila* have yielded significant cellular and molecular mechanistic insights into the cardioprotective functions of autophagy. In *Drosophila*, autophagy-related cardiac dysfunction has been closely associated with dysregulation of the mTOR/ULK1 signaling axis. Notably, pharmacological inhibition of mTORC1 reactivates ULK1 to enhance autophagic flux, and ameliorate cardiomyopathic phenotypes observed in *Lamin C* (LamC) mutants (Demir and Kacew, 2023; Hegedűs et al., 2014; Zhang et al.). These LamC mutants display compromised nuclear envelope integrity and disrupted nuclear–cytoplasmic communication, resulting in proteotoxicity and oxidative stress—classic features of impaired autophagy (Bhide et al., 2018; Chandran et al., 2019; Kirkland et al., 2023; Walker et al., 2023). Mutations affecting nuclear envelope components, such as LamC, interfere with the mTORC1–ULK1 axis, suppressing autophagic activity and leading to cardiac dysfunction. Treatment with rapamycin, an mTORC1 inhibitor, effectively restores autophagic flux and improves cardiac outcomes, underscoring the conserved role of mTOR signaling in cardiac homeostasis.

Crucial core autophagy-related genes *Atg1*, *Atg5*, and *Atg8* are indispensable for autophagosome formation and normal cardiac function. Upstream metabolic regulators of these genes through the AMPK and the PI3K/Akt pathway modulate mTORC1 activity to influence autophagic flux, linking energy sensing and growth signaling for cardiac maintenance and remodeling (Kirkland et al., 2023; Walker et al., 2023; Pai et al., 2023). These regulatory interactions form part of a broader metabolic network essential for adapting the postnatal heart to physiologic stress. Disturbances in this pathway leads to early postnatal death. Collectively, findings from *Drosophila* provide a robust genetic and biochemical foundation for understanding evolutionarily conserved autophagy mechanisms contributing to cardiomyocyte resilience in human cardiac disease. This cross-species paradigm reinforces the translational relevance of autophagy as a therapeutic target for mitigating cardiac disease arising from genetic mutations or environmental insults.

## Autophagy in viral and inflammatory cardiomyopathy: HIV as a model of immune-metabolic disruption

The pathogenesis of HIV-associated cardiomyopathy (HACM) underscores the detrimental consequences of impaired autophagy in the adult human heart. HIV infection affects chaperone-mediated autophagy (CMA) to cause cardiomyopathy in infected patients (collected in this Research Topic, Sun et al.). Dysregulation of CMA—critical for maintaining cardiomyocyte and macrophage mitochondrial integrity and immune homeostasis—leads to the accumulation of reactive oxygen species (ROS), activation of the inflammasome, and induction of pyroptosis (Gatica et al., 2022; Avula et al., 2021; Bulló et al., 2021; Morales et al., 2020; Abdel-Rahman et al., 2021; Ning et al., 2023; Li et al., 2022). In HIV infection and exposure to highly active antiretroviral therapy (HAART), mTOR signaling becomes hyperactivated, thereby downregulating ULK1 (unc-51 like autophagy activating kinase 1), a gene essential to autophagosome biogenesis. Suppression of ULK1 leads to reduced autophagy and further compromising cardiomyocyte survival in the setting of viral or drug injury (Sun et al.; Akbay et al., 2020; Crater et al., 2022; Singh et al., 2022). Pharmacological inhibition of mTORC1 can restore ULK1 activity and reinstate mitochondrial quality control, highlighting a promising therapeutic axis in autophagy-based cardiac interventions.

HIV-associated clonal hematopoiesis (CH)—propelled by somatic mutations in *Tet2*, *Dnmt3a*, and *Jak2*—further exacerbates inflammatory responses through impaired mitophagy and dysregulated macrophage signaling (Dharan et al., 2021; Bick et al., 2022; Wang et al., 2022). The pathological relevance of autophagic disruption in immune-mediated myocardial damage is reinforced by the observation that autophagy inhibition aggravates cardiac inflammation and slows adaptive remodeling. These mutations replicate the dysfunctional immune-metabolic axis previously observed in autophagy-deficient *Drosophila* models. Thus providing a mechanistic bridge linking immune dysregulation to cardiac injury in human disease. This evolutionary parallel accentuates the conserved role of autophagy in modulating inflammatory and metabolic homeostasis across species.

## Autophagy in metabolic stress and cardiac remodeling: maintaining homeostasis in metabolic stress

In models of obesity and diabetes, such as high-fat diet (HFD) combined with streptozotocin (STZ)-induced metabolic cardiomyopathy (MCM), autophagic activity is markedly suppressed due to insulin resistance-mediated hyperactivation of the mTORC1 signaling pathway (collected in this Research Topic, Zhou et al., 2025). This suppression compromises the clearance of dysfunctional mitochondria and promotes lipid overload and oxidative stress—key pathological features characteristic of diabetic heart disease.

Autophagy is a critical adaptive response in HFD/STZ-induced models of MCM that counteracts metabolic stress through multiple regulatory mechanisms. Specifically, autophagy alleviates lipid accumulation, fibrosis, and mitochondrial dysfunction by engaging the AMPK/mTOR/ULK1 and PINK1/Parkin-dependent signaling pathways (Madonna et al., 2023; Wang et al., 2017; Lin et al., 2021; Zhang et al., 2022; Elrashidy and Ibrahim, 2021). Selective autophagy processes—including mitophagy (via PINK1/Parkin), lipophagy, and ferritinophagy—play central roles in maintaining mitochondrial integrity, regulating lipid metabolism, and controlling intracellular iron levels. Disruption of these pathways results in lipotoxicity, ferroptosis, and impaired energy metabolism (Li et al., 2025). Both selective and non-selective autophagy work in concert to sustain myocardial homeostasis by clearing cytotoxic byproducts and preserving organelle function. Transcriptional regulators such as FOXO3, BNIP3, and TFEB, along with autophagy-related microRNAs like miR-34a, coordinate the expression of autophagy and lysosome-associated genes (Wang et al., 2021). Genetically engineered disruptions along the AMPK/mTOR/ULK1 and PINK1/Parkin-dependent signaling pathways in *Drosophila* induces the same autophagy failure and metabolic derangements described in human pathology (Zhang et al.). These findings indicate that these tightly regulated autophagy signaling networks are evolutionarily critical cross species, and that precise modulation of autophagic flux is crucial in the prevention and management of metabolic heart failure.

## Macrophage autophagy, clonal hematopoiesis, and inflammatory atherosclerosis: immune crosstalk in human cardiovascular disease

Macrophage autophagy—particularly CMA—plays a pivotal role in regulating lipid metabolism, controlling inflammation, and maintaining mitochondrial function (Li et al., 2025; Duan et al., 2022). Dysfunctional CMA contributes to the formation of foam cells, increased ROS accumulation, and activation of the NLRP3 inflammasome, thereby promoting pyroptotic cell death (Nussenzweig et al., 2015; Zhang et al., 2022; Díez-Díez et al., 2024; Yunna et al., 2020; Duewell et al., 2010). Deficient CMA impairs cholesterol efflux and facilitates the buildup of lipid droplets by hindering ABCA1-mediated cholesterol transport. This results in degraded lipid droplet-coating proteins such as PLIN2, which reinforces a cycle of vascular lipid overload and inflammation central to development of atherosclerosis.

Clonal hematopoiesis-associated mutations in genes such as *Tet2*, *Jaks*, and *Dnmt3a* have been shown to suppress mitophagy and shift macrophage cytokine profiles toward pro-inflammatory phenotypes (Jaiswal et al., 2017; Fuster et al., 2017; Zhao et al., 2025; Abplanalp et al., 2020). These mutations commonly accumulate in macrophages with age, illustrating how somatic changes in hematopoietic stem cells may affect macrophage function in an age related fashion. This progressive impairment of macrophage autophagic regulation promotes chronic vascular inflammation and senescence-associated atherosclerotic progression to accelerate atherogenesis with age. Importantly, restoration of CMA activity in macrophages—either through LAMP2A overexpression or pharmacological activation—has been shown to attenuate vascular inflammation and enhance plaque stability (Valdor and Martinez-Vicente, 2024; Fernández-Alvira et al., 2017).

## Tunneling nanotube (TNT) mediated autophagy in heart development

Tunneling nanotubes (TNTs) are well-established intercellular communication structures that play vital roles in a wide range of cellular processes, including signal transduction, metabolic exchange, and immune regulation (Polak et al., 2015; Rustom et al., 2004; Sowinski et al., 2008; Wang and Gerdes, 2015; Rainy et al., 2013; Lou et al., 2012). Originally identified as thin, actin-based cytoplasmic extensions that physically connect distant cells, TNTs have been recognized as essential mediators of direct intercellular communication, enabling the transfer of ions, proteins, RNA, and even organelles between connected cells. Their subsequent discovery within the context of cardiac development introduced a new dimension to the study of these dynamic microstructures (Goodman et al., 2019; Hulsmans et al., 2017; Zhou et al., 2018; Li et al., 2025; Wang et al., 2010; Ariazi et al., 2017). This emerging evidence suggests that TNTs may participate not only in cellular communication during homeostasis but also in orchestrating complex developmental events, such as those underlying heart morphogenesis.

In the developing heart, where multiple cell lineages interact to establish coordinated tissue architecture, the presence of TNT-like structures (TNTLs) offers a compelling explanation for how autophagy—a key intracellular degradation and recycling process—may be integrated into intercellular signaling networks. Autophagy has long been recognized as a mechanism through which cells maintain homeostasis and modulate communication with their neighbors. The potential coupling of TNT-mediated signaling with autophagic activity provides a novel mechanistic framework for understanding cardiac embryogenesis, particularly in regions where cells of dual origin, such as the endocardium and myocardium, interact. Notably, TNTs have been implicated in macrophage-mediated autophagic communication, influencing electrical coupling and metabolic coordination between mature cardiomyocytes (Rustom et al., 2004; de Rooij et al., 2017; Morrison and Scadden, 2014; Polak et al., 2015; Barutta et al., 2023; Ottonelli et al., 2022; Venkatesh and Lou, 2019). These findings collectively underscore the potential for TNTs to serve as structural and functional intermediaries in cardiac tissue organization and homeostasis.

**FIGURE 1:**
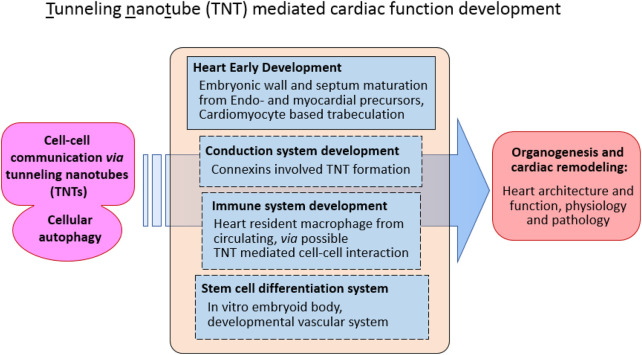
Function tunneling nanotube (TNT) in cellular communication during cardiac development.

Recent investigations have identified distinct TNT-like structures (TNTLs) within mouse embryonic hearts, further reinforcing their significance in early cardiac morphogenesis (Huynh, 2025; Miao et al., 2025; de la Pompa, J.L., 2025). The authors proposed that TNTLs localized within the cardiac jelly act as direct physical linkages between the endocardium and myocardium, facilitating the bidirectional exchange of signaling molecules and proteins between endocardial cells and cardiomyocytes. Using an *in vitro* co-culture system combined with fluorescently labeled cytoskeletal polymerization assays, these studies demonstrated that TNTL formation depends primarily on the polymerization of actin filaments rather than microtubule assembly (Miao et al., 2025; de la Pompa, J.L., 2025). This actin-dependent mechanism reflects the dynamic and adaptable nature of TNTLs, aligning with their proposed roles in modulating developmental signaling pathways (Figure 1).

Collectively, these discoveries provide new insight into how TNT-mediated autophagy and intercellular communication may contribute to cardiac development. By establishing physical and functional connections between endocardial and myocardial cells, TNTLs may help coordinate microstructural interactions and spatial patterning during cardiac trabeculation and ventricular wall formation. Understanding these processes could illuminate a previously underexplored layer of cardiac morphogenesis, offering new perspectives on how cellular connectivity and autophagic signaling jointly regulate the emergence of functional cardiac architecture.

## Cardiovascular aging and autophagy modulation *via* non-coding RNAs: senescence as a new frontier

Aging is the cumulative breakdown of cellular homeostasis. This is true in cardiovascular tissues where impaired autophagy exacerbates mitochondrial dysfunction, oxidative stress, and proteotoxicity over time. Non-coding RNAs (ncRNAs), including microRNAs (miRNAs) and long non-coding RNAs (lncRNAs), has recently been shown to regulate autophagy by modulating key signaling pathways, such as the previously discussed AMPK/mTOR and ULK1 pathways. Dysregulation of these ncRNAs contributes significantly to age-related cardiovascular decline (collected in this Research Topic, Scalabrin and Cagnin). Recent studies reveal that specific ncRNAs can enhance autophagic capacity and reduce markers of cellular senescence in endothelial cells and cardiomyocytes (Yang et al., 2017; Liang et al., 2020; Zhang et al.). From a therapeutic perspective, targeting ncRNAs to restore or optimize autophagic activity presents a promising strategy to delay cardiovascular aging and promote healthy lifespan extension.

## Conclusion and perspective

Autophagy is an evolutionarily conserved cellular function essential for life from embryogenesis to death. From the relatively simple heart tube of *Drosophila* to the anatomically complex four-chambered human heart, autophagy emerges as a central, evolutionarily preserved mechanism orchestrating cardiac development, physiological adaptation, healing, and age-related degeneration. Key molecular regulators of autophagy, such as mTOR, AMPK, ULK1, LAMP2A, and the PINK1/Parkin mitophagy pathway, and components of chaperone-mediated autophagy (CMA), are conserved across species and developmental stages. Normal autophagy is essential to normal cardiac development and functional recovery after myocardial injury. Abnormal autophagy has been implicated in the full spectrum of cardiac disease, from inherited genetic structural abnormalities to infectious cardiomyopathies like HIV-associated disease and cardiac disease from metabolic syndrome. The ubiquitousness of autophagy in embryonic development and diseases of pathologic cardiac remodeling represent a potential therapeutic target in which autophagy modulation could improve cardiac disease and recovery.
